# Correlation between intestinal flora disruption and protein–energy wasting in patients with end-stage renal disease

**DOI:** 10.1186/s12882-022-02762-2

**Published:** 2022-04-04

**Authors:** Jianguang Hu, Xiaoshi Zhong, Yan Liu, Jing Yan, Daoyuan Zhou, Danping Qin, Xiao Xiao, Yuanyuan Zheng, Luona Wen, Rongshao Tan, Pan Liang, Yun Liu

**Affiliations:** 1 Department of Nephrology, Guangzhou Red Cross Hospital, Medical College, Jinan University, Guangzhou, 510220 China; 2 Guangzhou Institute of Disease-Oriented Nutritional Research, Guangzhou, 510220 China

**Keywords:** 16S ribosomal DNA sequencing, Intestinal flora, Hemodialysis, End-stage renal disease

## Abstract

**Background:**

Different dialysis treatments may affect the composition and structure of the intestinal flora of dialysis-treated chronic kidney disease (CKD) patients. This study aimed to analyze the correlations between the different flora and the nutritional indexes and further explore the potential metabolic pathways in patients with CKD in end-stage renal disease (ESRD).

**Methods:**

Altogether, 102 patients with ESRD were recruited and categorized into the hemodialysis (HD) group (*N* = 49) and the peritoneal dialysis (PD) group (*N* = 53). Their biochemical indexes, anthropometric indicators, and inflammatory markers were determined. The total genomic DNA was extracted for 16S ribosomal DNA sequencing. Furthermore, bioinformatics analysis was employed for functional analysis.

**Results:**

Anthropometric indicators, including handgrip strength, mid-upper arm circumference, mid-upper arm muscle circumference, and body mass index, in the HD and PD groups showed a positive correlation with butyric acid-producing bacteria (*Rosella* and *Phascolarctobacterium*) and a negative correlation with conditional pathogens (*Escherichia* spp.). Meanwhile, the inflammatory markers, including high-sensitivity C-reactive protein and interleukin-6, were significantly higher in the PD-protein–energy wasting (PEW) group than in the PD-non-protein–energy wasting (NPEW) group; although they showed an increasing trend in the HD-PEW group, no significant difference was noted. *Rosella* was considerably scarce in the HD-PEW group than in the HD-NPEW group, whereas *Escherichia* was substantially more abundant in the PD-PEW group than in the PD-NPEW group. Compared with the HD group, the essential amino acid synthesis pathway, amino acid metabolism-related enzyme pathways, and aminoacyl-transfer RNA biosynthesis pathways were weakened in the PD group. Most carbohydrate metabolic pathways were weakened, although the tricarboxylic acid cycle was slightly enhanced. Concurrently, the fatty acid metabolism was enhanced, whereas fatty acid synthesis was weakened; the metabolic pathways of B vitamins were also weakened. These potential metabolic pathways of the various compounds released by intestinal flora showed a significant correlation with blood biochemical indexes, anthropometric indicators, and inflammatory markers.

**Conclusion:**

In patients with ESRD, different dialysis treatments affected the abundance of butyric acid-producing bacteria (*Rosella* and *Phascolarctobacterium*) and conditional pathogens (*Escherichia* spp.). Butyric acid-producing bacteria showed a positive correlation with PEW and showed a negative correlation with *Escherichia*. Improving the intestinal diversity and increasing the amount of butyric acid-producing bacteria, such as *Blautella, Faecococcus*, and *Phascolarctobacterium*, are potential therapeutic approaches to enhance protein–energy consumption in patients with ESRD.

## Introduction

Renal replacement therapies, such as hemodialysis (HD) and peritoneal dialysis (PD), play a critical role in extending the life span of patients with chronic kidney disease (CKD) in end-stage renal disease (ESRD) [1]. However, dialysis-induced complications can considerably affect the quality of life of patients. Among these complications, malnutrition contributes strongly to the prediction of a high hospitalization rate, low survival rate, and poor prognosis in patients with ESRD.

Protein–energy wasting (PEW) is a unique presentation of protein–energy undernutrition in people with kidney disease characterized by body protein catabolism that exceeds anabolism. Both low muscle mass and low fat are associated with high all-cause mortality in patients undergoing HD [2]. PEW is common in patients with maintained HD and continuous ambulatory PD [3, 4], with an incidence rate of 22.4–75% [5, 6]. Therefore, PEW prevention and treatment are particularly vital for the survival of patients with ESRD [4]. However, PEW still lacks a specific preventive measure, which is urgently required to improve the nutritional status and long-term prognosis of patients with ESRD who are undergoing dialysis.

The intestinal flora is essential for nutrient absorption, micronutrient synthesis, and drug metabolism. It can also synthesize various enzymes to enhance the digestive capacity and regulate energy balance [4]. Gehrig and Raman reported that a disordered intestinal flora is associated with undernutrition [7]. In addition, the structure of the intestinal flora is associated with nutritional status in children and patients with systemic sclerosis [8, 9]. Mice transplanted with microflora from malnourished infants exhibited a substantial decline in weight gain; some even became malnourished [10]. When a specific food was administered to malnourished children to reshape their intestinal flora, the intestinal flora showed significant improvement, eventually attaining the standard amount of intestinal flora in healthy children in the same region [7]. Therefore, improvement in the intestinal flora may enhance the nutritional status of individuals. Thus, the diversity, composition, and abundance of the intestinal microbiome; the effect of differentially expressed flora on the metabolism of major nutrients; and potential targets that may interfere with protein–energy consumption in patients with ESRD will be studied. PEW scores have recently been associated with intestinal dysbiosis in patients undergoing HD [11]. However, the relationship between the intestinal flora of patients undergoing PD and the occurrence of PEW remains unclear.

We previously showed that the composition and structure of the intestinal flora differ in patients with CKD who undergo different HD therapies [12]. Therefore, this study aimed to further analyze the nutritional status and metabolic functions of patients with ESRD undergoing different HD therapies.

## Methods

### Patient selection

We recruited patients diagnosed with ESRD in Guangzhou Red Cross Hospital and Panyu Central Hospital between 2017 and 2019. We then categorized them into two groups: HD group (*N* = 49) (age: 59.53 ± 10.57 years) and PD group (*N* = 53) (age: 57.7 ± 8.52 years). The ethics committee of the Guangzhou Red Cross Hospital approved this study (No. 2017–032-01), and all eligible patients provided written informed consent. The study adhered to the tenets of the Declaration of Helsinki and the Guidance on Sample Collection of Human Genetic Diseases by the Ministry of Public Health of China.

The inclusion criteria were as follows: 1) no intake of foods and drugs (e.g., including prebiotics, probiotics, and synbiotics) that are involved in the regulation of the intestinal flora in the past 6 months; 2) no administration of antibiotics, hormone, or immunosuppressive agents in the past 3 months; and 3) dialysis treatment for at least 6 months, with no changes in the dialysis mode and volume in the past 1 month. The exclusion criteria were as follows: 1) infection of the respiratory tract, digestive tract, or urinary tract; 2) type 1 or 2 diabetes mellitus; 3) gastrointestinal tumors or a history of abdominal organ surgery; or 4) liver disease or abnormal liver function.

### Blood biochemical test and inflammatory marker detection

We collected 8 ml of peripheral venous blood from each patient in the PD group on an empty stomach before the fluid change and from each patient in the HD group before the last dialysis. The blood levels of biochemical markers (e.g., blood urea nitrogen, serum creatinine, blood uric acid, hemoglobin, albumin, prealbumin, total cholesterol [TC]) and inflammatory factors (e.g., interleukin-6 [IL-6] and high-sensitivity C-reactive protein [hs-CRP]) were measured at Clinical Pathological Nutrition Institute of Guangzhou Red Cross Hospital.

### Anthropometric data measurement

Anthropometric data included height, weight, body mass index (BMI), handgrip strength (HGS), mid-upper arm circumference (MAC), mid-upper arm muscle circumference (MAMC), and triceps skinfold (TSF) thickness. Data in the HD group were measured after dialysis. These anthropometric data were measured as follows: 1) BMI was calculated using the following formula: BMI = weight (kg) / length (m^2^). 2) For the measurement of HGS, the non-arteriovenous fistula side limbs of the patient were measured thrice using the WCS-100 standard grip device developed by the State Sports General Administration; the maximum value was the final HGS value. 3) TSF thickness (mm) was obtained at the midpoint of the nondominant arm (between the acromial process and the olecranon), with the arm freely stretched along the body. 4) MAC was measured between the shoulder tip and the elbow tip (http://www.motherchildnutrition.org/early-malnutrition-detection/detection-referral-children-with-acute-malnutrition/muac.htm). 5) MAMC (cm) was calculated using the following formula proposed by Jelliffe: C2 = C1–3.14 × S, where C2 is the MAMC, C1 is the MAC, and S is the TSF thickness (cm) [13]. HGS, TSF, MAC, and MAMC were measured thrice, and the mean of these measurements was used as the final values.

### Genome 16S ribosomal DNA (rDNA) sequencing of the intestinal flora

As described in our previous study [12], the stool DNA test was extracted using a DP328 DNA extraction kit according to the manufacturer’s manual (Tiangen, Beijing, China). We qualitatively detected the total extracted genomic DNA by 1% agarose gel electrophoresis and determined its concentration using the Qubit® dsDNA HS Assay Kit (Thermofish, MA, USA). For the 16S ribosomal DNA (rDNA) V3 region, we used an upstream primer 338F and a downstream primer 534R for amplification and sequencing on the Illumina HiSeq2500 platform (Novogene, Beijing, China).

### 16S rDNA sequencing analysis of the intestinal flora

The operational taxonomic unit was compared using the RDP Classifier (v.2.2). Next, we used the Greengenes database for 16S bacterial and archaeal genome comparison, the Sliver database for fungal 18S sequences, and the UNITE database for fungal internal transcribed spacer sequences. The observed species index, Chao1 index, abundance-based coverage estimator index, Shannon index, Simpson index, and Good’s coverage index were selected to reflect the alpha diversity of the samples. Furthermore, we analyzed the third level Kyoto Encyclopedia of Genes and Genomes pathway (www.kegg.jp/kegg/kegg1.html) and abundance according to the different numbers of 16S rRNA copy numbers using PICRUSt [14–16].

### Statistical analysis

Quantitative data are expressed as mean ± standard deviation, and non-normally distributed data are expressed as median (interquartile range). For data with normal distribution and homogeneous variances, two study groups were compared using an independent-sample *t*-test; if the data did not meet the abovementioned conditions, Mann–Whitney *U* nonparametric test was used. The mean of multiple groups was compared using one-way analysis of variance analysis. For bivariate correlation, we employed Pearson or Spearman correlation analysis. All statistical data were analyzed using SPSS version 22.0 (SPSS Inc., Chicago, IL, USA), with a *p* value of < 0.05 considered statistically significant.

## Results

### Blood biochemical indexes, anthropometric indicators, and intestinal flora changes between the PEW and NPEW groups

In accordance with the PEW diagnosis criteria proposed by the International Society of Kidney Nutrition and Metabolism in 2008, we further divided the HD and PD groups into the HD-PEW group (*N* = 16), HD-non-PEW (NPEW) group (*N* = 33), PD-PEW group (*N* = 19), and PD-NPEW (*N* = 34) group. The levels of nutrition, inflammatory response, bacterial abundance, bacterial amino acid metabolic pathway, and the anthropometric indexes of each group were also measured.

The levels of albumin and prealbumin were higher in the HD-NPEW and PD-NPEW groups than in the HD-PEW and PD-PEW groups (*p* = 0.01). The levels of hs-CRP (*p* = 0.008) and IL-6 (*p* = 0.001) were significantly higher in the PD-PEW group than in the PD-NPEW group. Furthermore, HGS, MAC, MAMC, and BMI were significantly lower in the PEW groups than in the NPEW groups (*p* < 0.01). Regarding the functional analysis of the dominant bacteria, we found that branched-chain amino acid (BCAA) degradation and metabolic pathways were significantly enriched in the HD-PEW group than in the HD-NPEW group (*p* = 0.001). Regarding the abundance of dominant bacteria, *Roseburia* was significantly scarce in the HD-PEW group compared with the HD-NPEW group (*p* = 0.022), whereas *Escherichia* in the PD-PEW group was roughly thrice that in the PD-NPEW group (*p* = 0.022) (Table 1).

**Table 1:** Blood biochemical indexes, anthropometric indicators, and intestinal flora changes between the PEW and NPEW groups

Blood biochemical markers	HD (*N* = 49)		PD (*N* = 53)	
	PEW (*N* = 16)	NPEW (*N* = 33)	PEW (*N* = 19)	NPEW (*N* = 34)
Male:female	8:8	20:13	11:8	18:16
BMI (kg/m^2^)	21.13 ± 3.53	23.45 ± 2.76	20.27 ± 3.24	23.88 ± 2.92
Prealbumin (g/L)	0.23 ± 0.06	0.35 ± 0.10	0.21 ± 0.05	0.29 ± 0.08
Albumin (g/L)	32 ± 5.11	35.09 ± 4.53	20.3 ± 7.49	4.64 ± 5.08
hs-CRP	6.01 ± 2.48	4.7 ± 2.65	20.3 ± 7.49	4.64 ± 5.08
IL-6	7.15 ± 3.14	6.35 ± 3.42	21.7 ± 54.89	7.52 ± 6.78
TC	4.37 ± 0.9	4.33 ± 1.05	4.93 ± 1.27	5.19 ± 1.1
HGS (kg)	18.78 ± 4.74	29.30 ± 6.79	16.81 ± 4.67	21.04 ± 6.3
MAC (cm)	21.13 ± 2.60	24.05 ± 3	19.03 ± 2.98	21.78 ± 2.91
MAMC (cm)	20.62 ± 2.65	23.63 ± 2.99	18.48 ± 3.14	21.25 ± 2.98
Shannon index	2.55 ± 0.37	3.47 ± 0.33	1.42 ± 0.45	2.31 ± 0.72
Simpson index	0.17 ± 0.07	0.09 ± 0.08	0.25 ± 0.17	0.45 ± 0.16
Lysine biosynthesis	0.84 ± 0.09	0.88 ± 0.07	0.6 ± 0.09	0.45 ± 0.16
Histidine metabolism	0.63 ± 0.09	0.64 ± 0.093	0.41 ± 0.10	0.50 ± 0.106
Aromatic biosynthesis	0.84 ± 0.11	0.89 ± 0.08	0.66 ± 0.08	0.75 ± 0.11
BCAA biosynthesis	0.74 ± 0.08	0.78 ± 0.07	0.64 ± 0.08	0.68 ± 0.09
BCAA degradation	0.23 ± 0.05	0.18 ± 0.06	0.34 ± 0.09	0.28 ± 0.15
*Roseburia*	0.60 ± 1.13	2.35 ± 2.81	0.007 ± 0.018	0.299 ± 0.9
*Escherichia*	7.01 ± 12.23	2.59 ± 5.01	35.81 ± 29.46	13.58 ± 21.85
*Blautia*	2.58 ± 2.4	4.53 ± 3.92	0.54 ± 0.38	6.73 ± 8.15
*Phascolarctobacterium*	3.54 ± 2.76	3.44 ± 3.32	0.11 ± 0.31	1.15 ± 1.23
*Enterococcus*	0.09 ± 0.21	0.02 ± 0.11	11.11 ± 1.91	13.16 ± 3.02

*BCAA* branched-chain amino acid, *BMI* body mass index, *hs-CRP* high-sensitivity C-reactive protein, *HD* hemodialysis, *HGS* handgrip strength, *IL-6* interleukin-6, *MAC* mid-upper arm circumference, *MAMC* mid-upper arm muscle circumference, *NPEW* non-protein–energy wasting group, *PD* peritoneal dialysis, *PEW* protein–energy wasting group, *TC* total cholesterol, *TSF* triceps skinfold thickness

### Predicted metabolic pathways of dominant bacteria in the intestinal flora

Our previous study revealed that a disordered intestinal flora can severely affect the nutrition level of patients with CKD undergoing PD [12]. The present study further analyzed the effect of the dominant bacteria present in the intestinal flora on the metabolism of macronutrients and micronutrients.

**Fig. 1:**
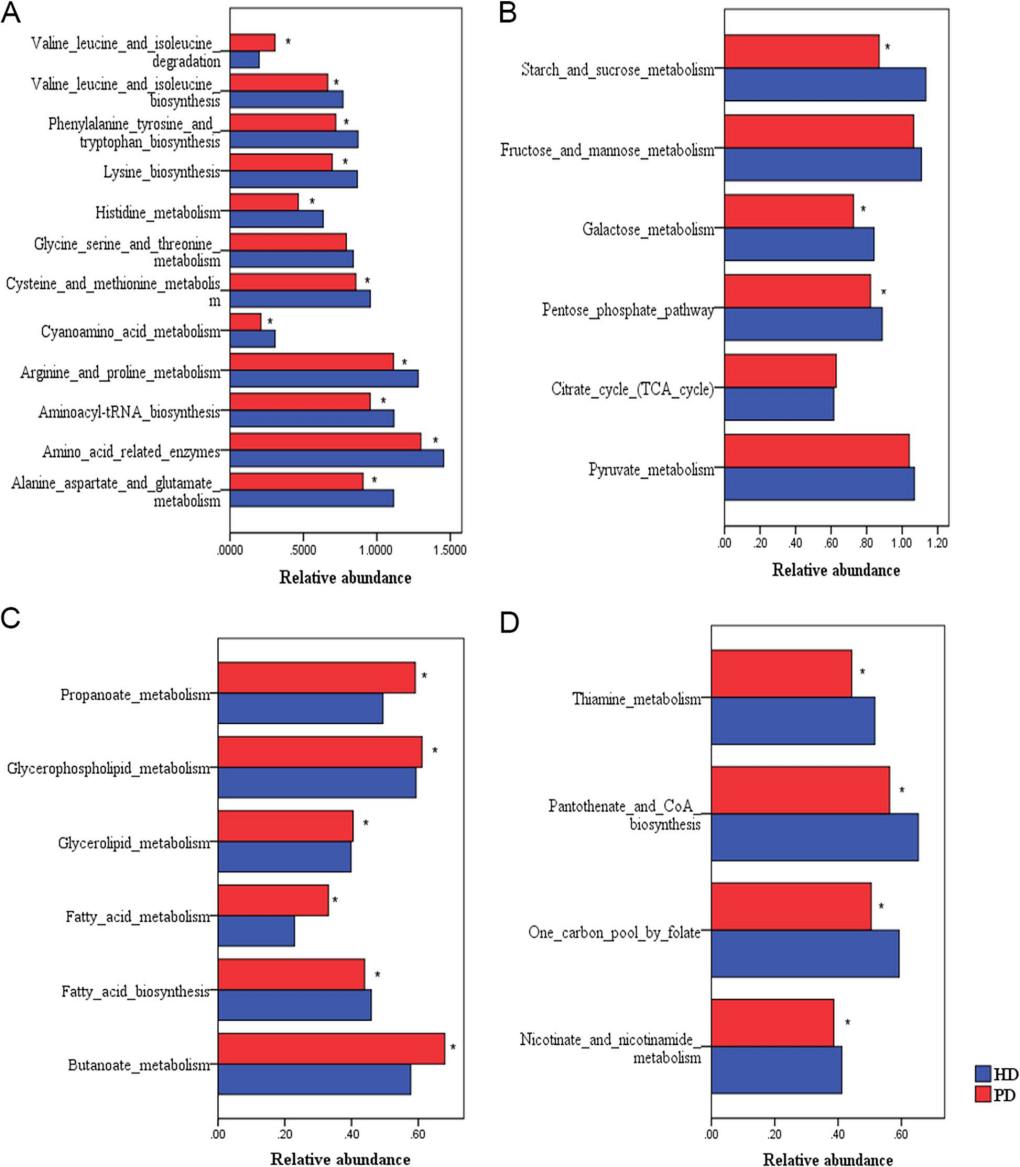
Predicted metabolic pathways of dominant microflora. **A**. Amino acid metabolism. Synthetic pathways and metabolic pathways were significantly reduced, whereas the valine, leucine, and isoleucine degradation pathways were significantly enhanced in the PD group compared with those in the HD group. **B**. Glucose metabolism. The tricarboxylic acid cycle was slightly increased, whereas the other metabolic pathways, including those of starch and sucrose metabolism, galactose metabolism, and pentose phosphate, were reduced in the PD group compared with those in the HD group. **C**. Lipid metabolism. The metabolisms of fatty acid, propionate, butyrate, glyceride, and glycerophospholipid were significantly enhanced, but the fatty acid synthesis pathway was significantly weakened in the PD group compared with those in the HD group. **D**. Vitamin metabolism. Nicotinate and nicotinamide metabolism (vitamin B3), pantothenic acid and coenzyme A biosynthesis (vitamin B5), one_carbon_pool_by_folate (vitamin B9), thiamine metabolism (vitamin B1), and other B vitamin metabolic pathways in the PD group were significantly weakened compared with those in the HD group

Regarding amino acid metabolism (Fig. 1A), the PD group had significantly lower incidence of synthetic pathways of essential amino acids (e.g., lysine, valine, leucine, isoleucine, phenylalanine, tyrosine, and tryptophan) than the HD group (*p* < 0.01). However, the metabolism of alanine, aspartic acid, and glutamic acid, and the metabolism of cysteine and methionine were significantly reduced in the PD group (*p* < 0.01). In terms of glucose metabolism (Fig. 1B), starch and sucrose, galactose, and pentose phosphate synthesis pathways were weaker in the PD group than in the HD group (*p* < 0.01). Regarding lipid metabolism (Fig. 1C), compared with the HD group, the PD group showed significantly enhanced metabolisms of fatty acid, propionate, butyrate, glyceride, and glycerophospholipid (*p* < 0.01) but showed a significantly weakened fatty acid synthesis pathway (*p* < 0.01). With respect to vitamin metabolism (Fig. 1D), the nicotinate and nicotinamide metabolism (vitamin B3), pantothenic acid and coenzyme A biosynthesis (vitamin B5), one carbon pool by folate (vitamin B9), thiamine metabolism (vitamin B1), and other B vitamin metabolic pathways were significantly weaker in the PD group than in the HD group (*p* < 0.01).

### Correlation between biochemical indicators and intestinal flora diversity

In both the HD and PD groups, the the serum levels of prealbumin showed a positive correlation with the Shannon index (r = 0.731 and r = 0.688) and a negative correlation with the Simpson index (r = − 0.59 and r = − 0.653) (*p* < 0.05), indicating that a higher bacterial diversity was accompanied by higher serum levels of prealbumin in both groups. In addition, a higher bacterial diversity in the HD group was accompanied by increased levels of blood uric acid (*p* < 0.05). Furthermore, the IL-6 levels in the serum in the PD group showed a negative correlation with the Shannon index (r = − 0.496, *p* = 0.001) and a positive correlation with the Simpson index (r = 0.518, *p* = 0.001); thus, the intestinal flora diversity in the PD group showed a negative correlation with inflammatory markers (Table 2).

**Table 2:** Correlation between biochemical indicators and intestinal flora diversity

Flora diversity		PAB		ALB		TC		UA		IL-6		CRP	
		r	*p*	r	*p*	r	*p*	R	*p*	r	*p*	r	*p*
HD	Shannon index	0.731	0.001	0.219	0.131	0.037	0.362	0.345	0.015	− 0.007	0.962	− 0.029	0.843
	Simpson index	−0.59	0.001	− 0.213	0.142	−0.042	0.772	−0.461	0.001	0.032	0.827	0.001	0.992
PD	Shannon index	0.688	0.001	0.230	0.097	0.111	0.430	0.068	0.630	−0.496	0.001	−0.304	0.027
	Simpson index	−0.653	0.001	−0.139	0.322	−0.079	0.574	−0.095	0.501	0.518	0.001	0.231	0.096

*ALB* albumin, *HD* hemodialysis, *CRP* C-reactive protein, *IL-6* interleukin-6, *PAB* prealbumin, *PD* peritoneal dialysis, *TC* total cholesterol, *UA* uric acid

### Correlation between biochemical markers and the dominant bacteria

The abundance of *Blautella*, *Escherichia*, *Enterococcus*, and *Salmonella* was closely associated with serum biochemical nutritional indicators in the HD group, among which *Escherichia* and *Enterococcus* showed a negative correlation with serum prealbumin (*p* < 0.05) and *Salmonella* showed a negative correlation serum albumin (*p* < 0.05). In the PD group, *Blautella*, *Escherichia*, and *Salmonella* were closely associated with serum biochemical nutritional indicators, among which *Escherichia* and *Salmonella* showed a negative correlation with serum prealbumin (*p* < 0.05). *Blautella* showed a positive correlation with serum prealbumin in both the HD and PD groups. Only the abundance of *Escherichia* in the PD group showed a positive correlation with inflammatory factors (i.e., IL-6 and hs-CRP) (*p* < 0.05). However, the relative abundance of the dominant bacteria and the inflammatory factors in the HD group showed no significant correlation (*p* > 0.05) (Table 3).

**Table 3:** Correlation between biochemical markers and the dominant bacteria

		PAB		ALB		IL-6		CRP	
		r	*p*	r	*p*	R	*p*	r	*p*
HD	*Escherichia*	−0.342	0.016	−0.026	0.861	0.195	0.180	0.150	0.304
	*Salmonella*	−0.128	0.382	−0.309	0.031	0.083	0.571	0.037	0.799
	*Enterococcus*	−0.363	0.026	−0.183	0.209	0.097	0.506	0.182	0.212
	*Blautia*	0.32	0.048	0.191	0.190	−0.027	0.852	− 0.008	0.958
PD	*Escherichia*	−0.397	0.003	−0.229	0.099	0.449	0.001	0.273	0.048
	*Salmonella*	−0.292	0.034	0.157	0.261	0.206	0.14	0.028	0.734
	*Enterococcus*	−0.146	0.298	0.108	0.441	0.022	0.873	0.028	0.840
	*Blautia*	0.382	0.005	0.156	0.264	0.218	0.116	−0.154	0.271

*ALB* albumin, *HD* hemodialysis, *CRP* C-reactive protein, *IL-6* interleukin-6, *PAB* prealbumin, *PD* peritoneal dialysis, *TC* total cholesterol, *UA* uric acid

### Correlation between biochemical markers and the predicted amino acid metabolic pathway

**Fig. 2:**
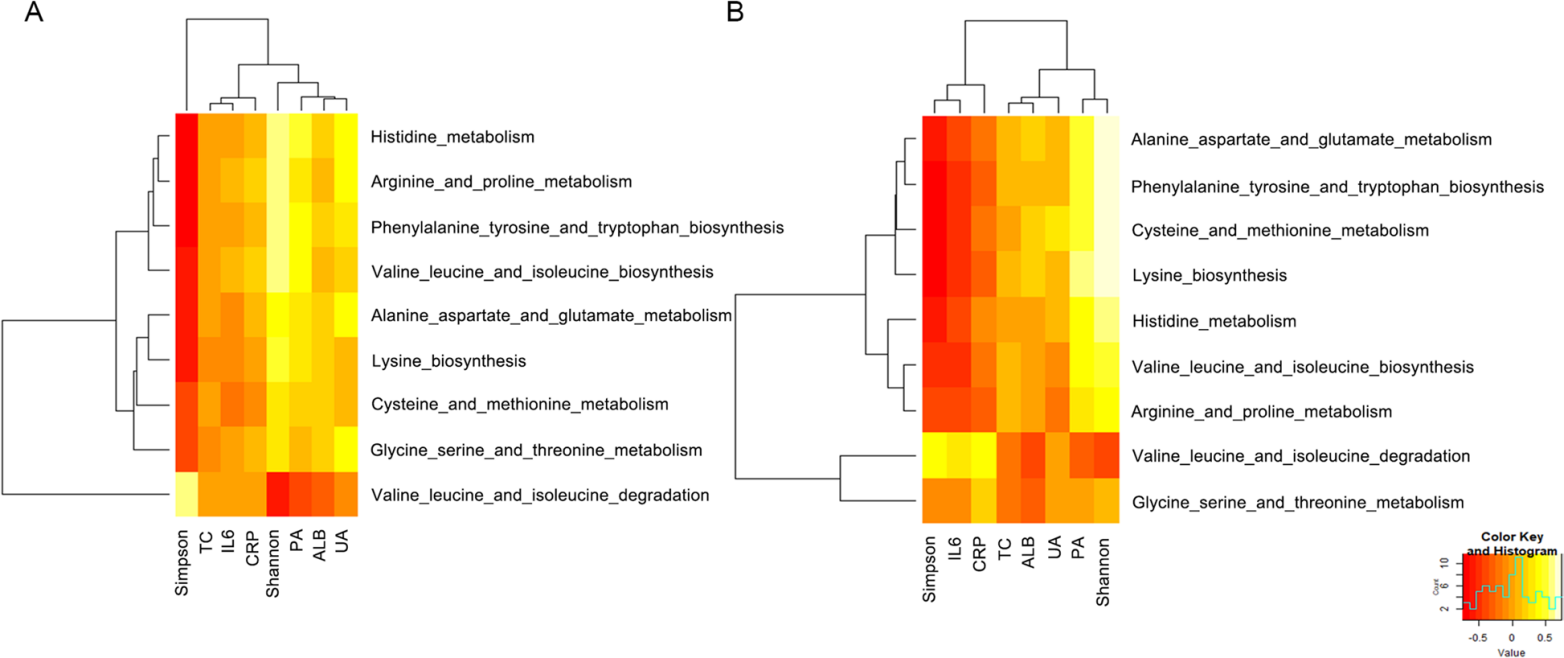
Correlation between biochemical markers and the predicted amino acid metabolic pathway of the HD group (**A**) and the PD group (**B**). The abscissa represents the flora diversity index and blood biochemical markers, and the ordinate represents the amino acid metabolism. The color scale bar ranges from − 5.0 to 5.0, with red, orange, and yellow representing low, medium, and high correlations, respectively

The levels of prealbumin in both the HD and PD groups showed a positive correlation with the predicted amino acid synthesis pathways (e.g., the biosynthesis of BCAA, lysine, aromatic amino acids, and histidine) (*p* < 0.01) and a negative correlation with the BCAA degradation pathway (*p* < 0.01). The level of IL-6 in both groups showed a negative correlation with the predicted amino acid synthesis pathways (e.g., the biosynthesis of BCAA, lysine, aromatic amino acids, and histidine) (*p* < 0.01) and a positive correlation with the BCAA degradation pathway (*p* < 0.05). Moreover, the level of hs-CRP in the PD group showed a negative correlation with the lysine biosynthetic pathway (*p* < 0.05) and a positive correlation with the BCAA degradation pathway (*p* < 0.05). No amino acid metabolic pathway correlated with inflammatory factors in the HD group (*p* > 0.05) (Table 4) (Fig. 2).

**Table 4:** Correlation between biochemical markers and the predicted amino acid metabolic pathway

	Amino acid metabolic pathway	CRP		IL-6		ALB		PAB	
HD (*N* = 49)	BCAA degradation	r	*p*	r	*p*	r	*p*	r	*p*
	Aromatic amino acid synthesis	0.164	0.26	0.335	0.013	0.140	0.338	−0.39	0.005
	Lysine synthesis	−0.078	0.592	0.035	0.89	0.243	0.092	0.39	0.005
	BCAA synthesis	0.007	0.96	−0.09	0.551	0.219	0.13	0.33	0.019
	Histidine metabolism	−0.01	0.949	0.041	0.778	−0.24	0.102	0.37	0.007
	Proline metabolism	0.171	0.241	0.057	0.695	0.143	0.325	0.33	0.019
	G/aspartate/glutamate metabolism	0.076	0.602	−0.06	0.680	0.221	0.112	0.31	0.029
	Amino acid metabolism pathway	−0.249	0.072	−0.462	0.001	0.036	0.789	0.372	0.016
PD (*N* = 53)	BCAA degradation	−0.267	0.054	−0.499	0.001	0.124	0.377	0.493	0.001
	Aromatic amino acid synthesis	− 0.335	0.014	−0.524	0.001	0.243	0.08	0.602	0.001
	Lysine synthesis	−0.148	0.291	−0.439	0.001	0.013	0.925	0.43	0.001
	BCAA synthesis	0.42	0.002	0.316	0.021	−0.355	0.009	− 0.329	0.016
	Histidine metabolism	−0.242	0.081	−0.454	0.001	0.185	0.184	0.525	0.001
	Proline metabolism	−0.234	0.091	−0.387	0.004	0.169	0.225	0.453	0.001
	G/aspartate/glutamate metabolism	−0.244	0.078	−0.419	0.002	0.043	0.761	0.323	0.018

*ALB* albumin, *BCAA* branched-chain amino acid, *CRP* C-reactive protein, *HD* hemodialysis, *IL-6* interleukin-6, *PAB* prealbumin, *PD* peritoneal dialysis, *TC* total cholesterol, *UA* uric acid

### Correlation between the dominant bacteria and the predicted amino acid metabolic pathway

**Fig. 3:**
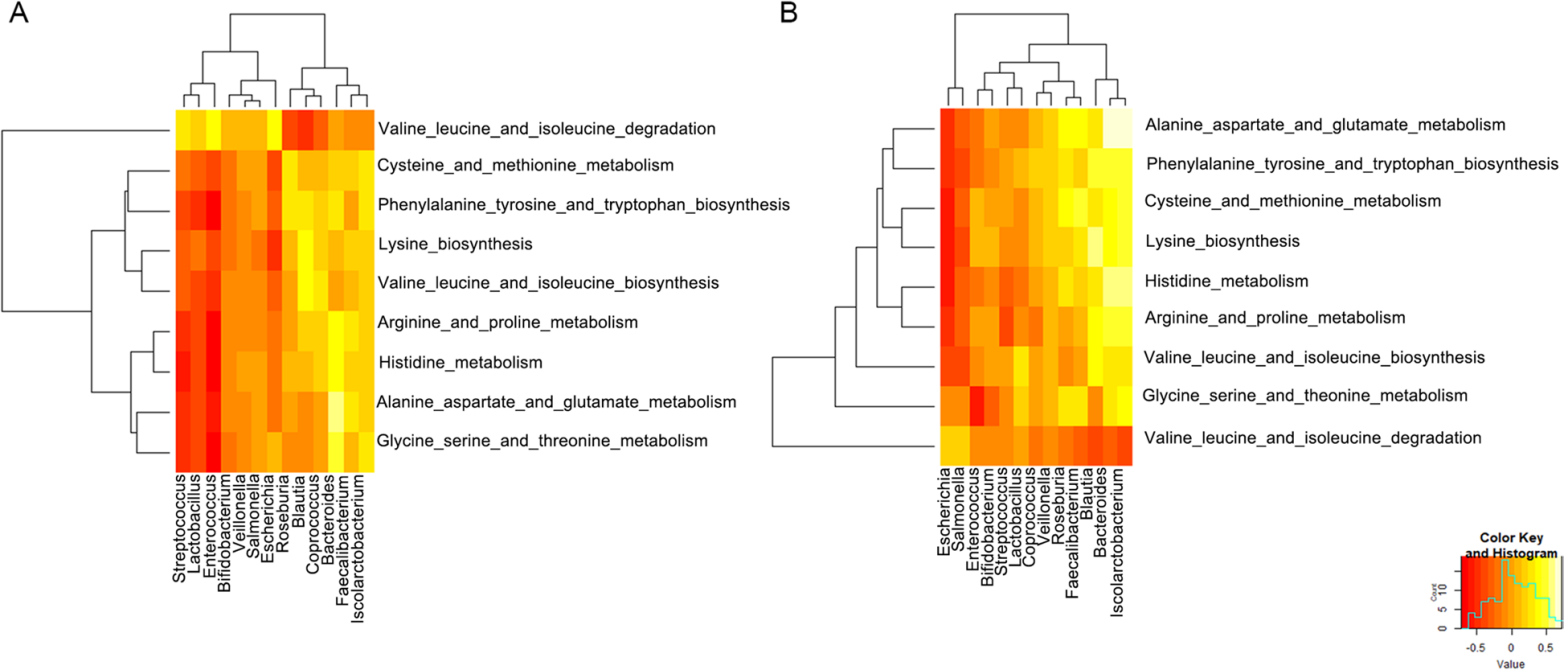
Correlation between dominant bacteria and the predicted amino acid metabolic pathway of the HD group (**A**) and the PD group (**B**). The color scale bar ranges from − 5.0 to 5.0, with red, orange, and yellow, representing low, medium, and high correlations, respectively

For further correlation analysis between the dominantly expressed bacteria and the predicted amino acid metabolic pathway, the abundance of *Escherichia* in both groups showed a negative correlation with the synthesis of BCAAs, aromatic amino acids, lysine, and histidine (*p* < 0.05) and a positive correlation with BCAA degradation (*p* < 0.05). In addition, *Enterococcus* in the HD group and *Salmonella* in the PD group showed a negative correlation with the amino acid synthesis pathway (*p* < 0.05) and a positive correlation with the amino acid degradation pathway (*p* < 0.05). The abundance of butyric acid-producing bacteria, such as *Bacteroides*, *Blautia*, *Rosella*, and *Phascolarctobacterium*, showed a positive correlation with the synthesis of BCAAs, aromatic amino acid, lysine, and histamine and a negative correlation with BCAA degradation pathways in both groups (*p* < 0.05). The abundance of *Escherichia* and *Salmonella* in the PD group showed a negative correlation with the synthesis of BCAAs, aromatic amino acids, lysine, and histidine (*p* < 0.05); in contrast, *Bacteroides*, *Blautia*, *Roseburia*, and *Phascolarctobacterium* showed a positive correlation (*p* < 0.05), but they showed a negative correlation with BCAA degradation pathways (*p* < 0.05) (Table 5) (Fig. 3).

**Table 5:** Correlation between the dominant bacteria and the predicted amino acid metabolic pathway

		BCAA synthesis		BCAA degradation		Aromatic amino acid synthesis		Lysine synthesis		Histidine synthesis	
		r	*p*	r	*p*	r	*p*	r	*p*	r	*p*
HD (*N* = 49)	*Escherichia*	−0.35	0.014	0.49	0.021	−0.33	0.021	−0.54	< 0.001	−0.22	0.123
	*Roseburia*	0.05	0.727	−0.41	0.003	0.30	0.034	0.124	0.398	0.082	0.577
	*Blautia*	0.43	0.002	−0.53	< 0.001	0.34	0.018	0.472	0.001	0.074	0.611
	*Bacteroides*	0.03	0.843	−0.04	0.808	0.34	0.017	0.166	0.253	0.42	0.003
	*Phascolarctobacterium*	0.30	0.027	−.035	0.009	0.52	< 0.001	0.484	< 0.001	0.534	< 0.001
	*Coprococcus*	0.34	0.010	−0.32	0.026	0.29	0.042	0.269	0.061	0.205	0.159
	*Enterococcus*	−0.59	< 0.001	0.48	< 0.001	−0.77	< 0.001	−0.49	< 0.001	−0.87	< 0.001
PD (*N* = 53)	*Escherichia*	−0.43	0.002	0.22	0.114	−0.05	< 0.001	−0.60	< 0.001	−0.58	< 0.001
	*Salmonella*	−0.36	0.009	0.15	0.299	−0.41	0.002	−0.40	0.003	−0.31	0.022
	*Roseburia*	0.07	0.627	0.23	0.101	0.28	0.036	0.195	0.162	0.282	0.041
	*Blautia*	0.43	0.001	−0.38	0.006	0.44	0.001	0.564	< 0.001	0.315	0.022
	*Bacteroides*	0.28	0.041	−0.31	0.023	0.49	< 0.001	0.411	0.002	0.601	< 0.001
	*Phascolarctobacterium*	0.31	0.027	−0.35	0.009	0.51	< 0.001	0.484	< 0.001	0.534	< 0.001

### Correlation between anthropometric indicators and intestinal flora diversity

Anthropometric indicators, including HGS, MAC, MAMC, and BMI, showed a positive correlation with the Shannon index (*p* < 0.05) and a negative correlation with the Simpson index (*p* < 0.05) in both the HD and PD groups (Table 6).

**Table 6:** Correlation between anthropometric indicators and flora diversity

Flora diversity		TSF		HGS		MAC		MAMC		BMI	
		r	*p*	r	*p*	r	*p*	r	*p*	r	*p*
HD (*N* = 49)	Shannon index	0.117	0.424	0.559	< 0.001	0.353	0.013	0.427	0.002	0.367	0.016
	Simpson index	−0.11	0.434	−0.440	0.002	− 0.278	0.053	−0.313	0.029	−0.418	0.009
PD (*N* = 51)	Shannon index	0.033	0.814	0.598	< 0.001	0.567	< 0,001	0.562	< 0.001	0.533	0.001
	Simpson index	−0.030	0.829	− 0.503	< 0.001	− 0.518	< 0.001	− 0.531	< 0.001	− 0.421	0.01

*BMI* body mass index, *HD* hemodialysis, *HGS* handgrip strength, *MAC* mid-upper arm circumference, *MAMC* mid-upper arm muscle circumference, *PD* peritoneal dialysis, *TSF* triceps skinfold thickness

### Correlation analysis between anthropometric indicators and the dominant bacteria

Anthropometric indicators, including HGS, MAC, MAMC, and BMI, showed a positive correlation with the butyric acid-producing bacteria *Rosella* and *Coprococcus* (*p* < 0.05) and a negative correlation with the pathogenic bacteria *Escherichia* spp. (*p* < 0.05) in both the HD and PD groups. The anthropometric indicators HGS, MAC, MAMC, and BMI showed a positive correlation with *Coccus* spp. in the HD group (*p* < 0.05) and a negative correlation with *Salmonella* in the PD group (*p* < 0.05) (Table 7).

**Table 7:** Correlation analysis between anthropometric indicators and the dominant bacteria

		TSF		HGS		MAC		MAMC		BMI	
		R	*p*	r	*p*	r	*p*	r	*p*	r	*p*
HD (*N* = 49)	*Escherichia*	−0.013	0.385	−0.388	0.006	−0.28	−0.051	−0.265	0.066	−0.46	0.012
	*Roseburia*	0.016	0.499	0.41	0.003	0.287	0.045	0.285	0.047	0.42	0.004
	*Phascolarctobacterium*	0.014	0.992	0.343	0.016	0.137	0.348	0.123	0.399	0.344	0.018
	*Coprococcus*	0.099	0.499	0.410	0.003	0.370	0.009	0.424	0.002	0.418	0.005
PD (*N* = 51)	*Escherichia*	−0.094	0.505	−0.212	0.127	−0.343	0.012	−0.361	0.008	−0.35	0.006
	*Roseburia*	0.012	0.932	0.292	0.034	0.273	0.048	0.251	0.07	0.26	0.06
	*Phascolarctobacterium*	0.024	0.864	0.399	0.003	0.44	0.001	0.421	0.002	0.444	0.001
	*Coprococcus*	0.024	0.864	−0.33	0.016	−0.330	0.016	−0.318	0.02	−0.32	0.013

*BMI* body mass index, *HD* hemodialysis, *HGS* handgrip strength, *MAC* mid-upper arm circumference, *MAMC* mid-upper arm muscle circumference, *PD* peritoneal dialysis, *TSF* triceps skinfold thickness

### Relationship between anthropometric indicators and the predicted amino acid metabolic pathways

In both groups, the anthropometric indicators HGS, MAC, MAMC, and BMI showed a positive correlation with the synthesis and metabolic pathways of aromatic amino acids, BCAAs, and lysine (*p* < 0.05) and a negative correlation with the BCAA degradation pathway (*p* < 0.05) (Table 8).

**Table 8:** Relationship between anthropometric indicators and the predicted amino acid metabolic pathways

Amino acid metabolism		HGS		MAC		MAMC		BMI	
		r	*p*	r	*p*	r	*p*	r	*p*
HD (*N* = 49)	Aromatic biosynthesis	0.458	< 0.001	0.37	0.009	0.382	0.007	0.33	0.017
	BCAA biosynthesis	0.37	0.009	0.347	0.015	0.93	0.005	0.448	0.005
	Lysine biosynthesis	0.389	0.006	0.34	0.017	0.315	0.027	0.335	0.018
	BCAA degradation	−0.485	< 0.001	−0.441	< 0.001	−0.12	0.399	−0.455	0.001
PD (*N* = 51)	Aromatic biosynthesis	0.512	< 0.001	0.471	< 0.001	0.448	0.001	0.456	0.001
	BCAA biosynthesis	0.36	0.008	0.345	0.016	0.953	0.005	0.488	0.004
	Lysine biosynthesis	0.42	0.002	0.488	< 0.001	0.489	< 0.001	0.466	< 0.001
	BCAA degradation	−0.37	0.009	−0.347	0.015	−0.93	0.005	−0.55	0.001

*BMI* body mass index, *BCAA* branched-chain amino acid, *HD* hemodialysis, *HGS* handgrip strength, *MAC* mid-upper arm circumference, *MAMC* mid-upper arm muscle circumference, *PD* peritoneal dialysis

## Discussion

This study analyzed the correlation between the intestinal flora and nutritional indicators in patients receiving different dialysis treatments. We specifically examined the correlation between the intestinal flora of patients undergoing HD and PD with PEW complications and nutritional indicators, as well as the predicted changes in the metabolic pathways that may be caused by changes in the intestinal flora.

The diagnostic criteria for PEW include blood biochemical and nutritional indicators (e.g., albumin, prealbumin, and cholesterol) and the anthropometric data (e.g., MAC, MAMC, HGS, BMI) [13, 17]. Serum prealbumin is closely associated with the protein metabolism in the body. Hypoproteinemia strongly predicts mortality in patients with ESRD [18]. The muscle is the largest protein storage organ in the human body, indicating the nutritional status of the body. Clinically, anthropometric data are often used to assess muscle capacity. Our findings indicated that serum levels of albumin and prealbumin in NPEW groups were higher than those in PEW groups, which is consistent with the findings of the previous study [11]. In addition, butyric acid-producing bacteria, such as *Roseburia* and *Phascolarctobacterium,* showed a positive correlation with HGS, MAC, MAMC, and BMI in dialysis patients, whereas the conditional pathogen *Escherichia* showed a negative correlation with these anthropometric indicators. *Coprococcus* only showed a positive correlation with HGS, MAC, MAMC, and BMI in HD group. Butyric acid-producing bacteria include *Roseburia*, *Phascolarctobacterium*, and *Coprococcus*. The main metabolite of butyric acid-producing bacteria is a short-chain fatty acid, which is essential for maintaining the integrity of the colonic mucosa and promoting anti-inflammation [19]. In the mouse intestine, a decrease in the abundance of butyric acid-producing bacteria reduced oxygen consumption by intestinal mucosal epithelial cells and promoted the growth of facultative anaerobes, including *Salmonella* [20]. Disruption of the dynamic balance between pathogenic and beneficial bacteria in the intestine can increase intestinal permeability, translocate bacteria and endotoxins in the intestine, and ultimately induce systemic inflammation [21, 22]. *Escherichia* is a commensal-type bacteria in the human intestinal tract that is capable of causing diseases. In mice, exposure to *Escherichia* can result in gastrointestinal inflammation and anxiety via the hypothalamic–pituitary–adrenal axis [23, 24]. In children, accumulation of *Escherichia* is associated with diarrhea, gastrointestinal inflammation, and diarrhea-induced chronic malnutrition [25, 26]. Furthermore, intestinal flora is essential in maintaining the quality and function of skeletal muscles. Shawon et al. transplanted the intestinal flora of ordinary experimental mice into the intestine of sterile mice and discovered that the muscle mass and function of sterile mice were significantly enhanced [27]. The “gut–muscle axis” connects the cross-talk between the intestinal flora and skeletal muscles [28]. Therefore, the decrease in butyric acid-producing bacteria in the intestinal tract of patients undergoing dialysis can induce intestinal inflammation and affect intestinal nutritional function. In fact, patients with PEW undergoing HD reportedly have a reduced abundance of butyric acid-producing bacteria, indicating that the decrease in butyric acid production is associated with PEW diseases [11]. Our study showed that the abundance of *Roseburia* was significantly scarce in the HD-PEW group and the abundance of *Phascolarctobacterium* was significantly greater in the PD-PEW group than in the NPEW group. Therefore, the decrease in *Roseburia* and *Phascolarctobacterium* and the increase in *Escherichia* in patients with PEW could be associated with intestinal permeability influence, inflammation increase, and nutritional imbalance influence. Thus, follow-up experimental evidence is required.

The concept of “microinflammation–malnutrition,” which was first proposed by Stenvinkel et al. in 1999, states that malnutrition and inflammation often coexist and act as a cause and effect for each other [29]. As treatment time increases, the nutritional status of patients with ESRD deteriorates, and most microinflammatory indicators show a negative correlation with nutritional indicators [11]. Based on our findings, malnutrition and microinflammation are major contributors to the poor prognosis of patients with ESRD. Furthermore, the PD-PEW group had significantly higher levels of microinflammatory markers, hs-CRP, and IL-6 than the NPEW group [30]. This could be because long-term retention of glucose from the peritoneal dialysate in the abdominal cavity of patients receiving PD alters the intestinal environment, resulting in the proliferation of *Escherichia coli* and other bacteria, increased intestinal mucosal permeability, and subsequently, microinflammation.

The intestinal flora can affect the nutritional status of the host through metabolites. The intestinal flora and its metabolites participate in the metabolism of the three major types of nutrients, i.e., carbohydrates, proteins, and fats [31]. They are involved in the occurrence and development of malnutrition by affecting the energy metabolism of the host and aggravating the microinflammatory response. The mechanism of protein–energy consumption in patients with ESRD has been widely explored [32, 33]; however, the possible relationship between changes in the intestinal flora and protein–energy consumption remains poorly elucidated. We showed that compared with the NPEW group, the PEW group had enhanced metabolic pathways for BCAA degradation. Besides assisting in the regulation of protein metabolism, the synthesis of valine, leucine, isoleucine, and all essential amino acids can help the body in strongly resisting muscle nutrient loss and reducing the decomposition and damage of muscle protein [34]. Specifically, leucine is the main driving force of muscle protein synthesis [35]. Therefore, the enhanced BCAA degradation in patients with PEW may explain the decline in nutritional levels. An imbalance in amino acid metabolism results in muscle protein degradation, which may eventually result in the occurrence and development of protein–energy consumption.

Compared with the HD group, the PD group exhibited a decline in the essential amino acid synthesis, amino acid metabolism-related enzyme, and aminoacyl-transfer RNA biosynthesis pathways; with the exception of a slight increase in the tricarboxylic acid cycle pathway, the other sugar metabolic pathways were weakened. Lipid metabolic pathways such as fatty acid metabolism increased, whereas fatty acid synthesis decreased, and B vitamin metabolic pathways were weakened. Thus, PD has a stronger energy metabolism in the peritoneum than HD, and it weakens nutrient synthesis, putting patients at risk of micronutrient loss, especially B vitamins. This event could be explained by the loss of peritoneal dialysate protein after injecting the dialysate into the abdominal cavity and the preferential use of glucose in the dialysate, which inhibits the metabolic pathways of monosaccharides and glycans. This aspect still requires further research. Furthermore, the difference in energy metabolism between patients receiving PD and HD indicates that adopting different nutritional management strategies for patients receiving HD and PD may be required in clinical treatment so as to provide gut microbiological evidence.

The main limitation of this study is the lack of experiments to verify dietary intake. Therefore, future studies will require additional multicenter research and intervention studies for the analysis of dietary intake. Despite this limitation, our findings provide evidence regarding the diversity and components of intestinal flora that should be taken into consideration to improve protein–energy consumption in patients with ESRD.

## Conclusion

Butyric acid-producing bacteria showed a positive correlation with PEW and a negative correlation with *Escherichia*. Improving the diversity of the intestinal flora and increasing the abundance of butyric acid-producing bacteria, such as *Blautella*, *Faecococcus*, and *Phascolarctobacterium*, may be a novel means of improving protein–energy consumption in patients with ESRD. A specific mechanism at the molecular level should be investigated in future research.

## Data Availability

The datasets generated during the current study are available in the NCBI [Accession number: PRJNA813526].

## Abbreviations

CKD: Chronic kidney disease
HD: Hemodialysis
PD: Peritoneal dialysis
HGS: Handgrip strength
MAC: Mid-upper arm circumference
MAMC: Mid-upper arm muscle circumference
TSF: Triceps skinfold
BMI: Body mass index
PEW: Protein energy wasting
ESRD: End-stage renal disease
OUT: Operational taxonomic unit
IL-6: Interleukin-6
CRP: C reactive protein
